# Medical Items and News of Interest to the Physician

**Published:** 1877-10

**Authors:** 


### For the Use of Physicians.

CULLED FROM THE STANDARD MEDICAL JOURNALS OF THE WORLD.

NOTE.—These formulæ are intended only for the regular practitioner of medicine, and should be employed only by him, or under his immediate supervision.

**Chloral Hydrate**

Is said to be growing in favor as a local anæsthetic and stimulant in cancerous and other painful ulcers. The solution may vary from twenty grains to a drachm, to the ounce.

**Dr. Dublanchet,**

Formerly house-surgeon in one of the hospitals in Paris, died recently of diphtheria after less than forty-eight hours illness. The disease was contracted while attending to a child that was suffering from the croup.

**The Plague.**

In consequence of the extension of the plague to Bagdad and in Messopotamia, the Civil Governor of Algeria recently gave notice to the Mohammedans in his jurisdiction, that no more pilgrimages to Mecca will be allowed for the present. The interdict is not permanent, but will be raised as soon as circumstances will permit.

**Anthrax Maligna.**

We hear from *Le Temps* that an epidemic of carbuncular fever has broken out in New Caledonia, particularly in the Isle of Pines, and has caused great ravages among the inhabitants, as well as among animals. The flies appear to be the most active agents in spreading the contagion. The epidemic is on the increase; at the date of the report the number of victims was already above sixty.

**While there is Life there is Hope.**

Professor Esmarch, of Kiel, at the late Congress of German surgeons, drew attention to the fact that we are not warranted in dismissing every case of cancer as incurable where an operation cannot be performed. He recalled the history of a lady, who, when declared incurable, took arsenic, on purpose to destroy her life. She failed in her design, but cured her disease. He adduced, with illustrative drawings, a large number of patients the subjects of cancer or epithelioma, or of diseases indistinguishable from them, where large doses of iodide of potassium or of arsenic had cured the apparently hopeless malady.

**Feeling the Pulse by Telegraph.**

Some months ago Dr. Upham, of Salem, Mass., in order to explain to his audience the variations of pulse in certain diseases, caused the lecture-room to be placed in telegraphic communication with the city hospital of Boston, distant fifteen miles, and by means of special apparatus the various pulse-beats were exhibited by a vibrating ray of magnesium-light upon the wall. These experiments have lately been repeated at Paris with success.—*New Remedies.*

**Photographs of Bacteria.**

The photographs of bacteria, which were prepared by Dr. Koch, of Wollstein, already famous from his investigations on anthrax, and sent by him to Professor Ferd. Cohn, have created a sensation in medical circles in Breslau. Dr. Koch has, after patient study, discovered a method of taking photographs, which not only confirm previous observations on these important organisms, but, as the photographic plate is more sensitive than the retina of the eye, also render manifest some hitherto unknown peculiarities. The photographs so far exhibited show specimens of bacteria, bacilla, and spirilla, and of many hitherto unknown variety of spores.

**Mustard Paper.**

The preparation of paper sinapisms requires two indispensable conditions: 1. The employment of powdered mustard *entirely* deprived of all fatty matter. 2. The use of an adhesive liquid, which contains neither water nor alcohol, nor resin, nor fatty matters.

Powdered mustard is deprived of its fat or oil by means of carbon disulphide, or petroleum benzine. The adhesive liquid consists of a solution of four or five parts of caoutchouc in one hundred parts of a mixture of carbon disulphide and petroleum benzine. By means of a plaster-spreading apparatus a uniform coat of this solution is applied to paper. As the latter passes out from under the apparatus, a sieve containing the powdered mustard is shaken over it, which latter is fixed by the adhesive coat and firmly retained after the evaporation of the volatile liquids in a warm place. The application of the powdered mustard must be properly regulated according to the speed with which the machine delivers the coated paper. [ This is a very simple matter, when using the common small hand-machines. ]

The paper is cut into pieces of convenient size, and needs only to be wetted to be ready for use. *New Remedies.*

**A Turpentine Bath.**

We have seen many notices of most extraordinary kinds of baths for the removal of disease, but the latest and oddest is that of placing a patient in a box containing an atmosphere saturated with terebinthinate vapors. Good for Rheumatism!—*Druggists' Circular.*

**Citric Acid in Diphtheria.**

Dr. Caspari writes to the *Deutsche Med. Wochenschrift*, that he has treated successfully over forty cases of diphtheria by using locally (with the spray and brush) slightly diluted citric acid. Several of these cases had resisted treatment by salicylic and carbolic acid. Appropriate constitutional treatment was, of course, combined with the local.

**Lacto-Phosphate of Lime as a Tooth-Filling.**

The treatment of exposed dental pulps and sensitive dentine is the subject of a paper by Junius E. Cravens, D. D. S. The lacto phosphate of lime is applied to the exposed pulp, which is carefully sealed and left undisturbed for several weeks. On removing the coverings a new bone is found, its surface continuous with that of the formerly soft dentine, and the sensibility being even below the normal degree. Oxy-chloride of zinc and other substances, however, may produce like results.

**Transparent Gout-Paper.**

Tissue-paper is covered with a solution of 1 part of amber-lac and 6 parts of benzoin; when dry the following is applied: Euphorbium, 30 parts; cantharides, 15 parts; stronger alcohol, 300 parts; digest three days, filter, and add Venice-turpentime, resin, and pitch, of each 15 parts.

If necessary, this solution may be diluted with more strong alcohol, and should be applied with a broad brush.

Another formula is: Cantharides 15, euphorbium 4, strong alcohol, 240 parts; digest a few days, filter, and add pitch 180; dissolve with a gentle heat, and add Venice turpentine 6 parts, and enough tar to color the mixture brown. This is spread two or three times upon tissue-paper by means of a sponge, and when dry, the back of the paper is coated with oil of lavender.—(*Pharm. Zeit.*)

**Ergot in Atony of the Bladder.**

Prof. von Langenbeck, at a meeting of the Berlin Medical Society, stated that in atony of the bladder, associated with enlarged prostate, in elderly men, in which the organ is never completely emptied of urine, he had lately tried the hypodermic injection of ergotin with most surprising results. In three cases, the contractile power of the bladder was at once increased so as to enable the patient to discharge additional urine, and in a few days it had so augmented that very little urine was left behind. After one or two injections the improvement was considerable, and even a diminuation in the size of the prostate seemed to have ensued. Dr. Israel said that he had derived the same benefit from the employment of ergotin, and referred to the case of a patient who was thus enabled to hold his water for three hours, whereas before he voided it every ten minutes.—*New Remedies.*

**Lotions for Urticaria.**

Prof. Hardy, of Paris, recommends the following lotions to be applied several times daily:

℞

Chloroformi, 10 parts.
Olei amygdal. dulc., 30 parts. M.

In obstinate cases, he gives:

Hydrarg. chlor. corros., $\frac{1}{10}$ to $\frac{1}{7}$ parts.

Alcoholis, 10 parts.

Aq. destill. 90 parts. M.

He also gives internally alkaline medicines, and, if necessary, arsenic.

**Hemorrhoids.**

Dr. Riviere, in the *Gazette des Hopitaux*, December 16th, states that he has had fifteen cases which were most conclusive as to the value of podophyllin in hemorrhoids. Among the constipated patients, for whom he prescribed podophyllin, many suffered from hemorrhoids, which he attributed to permanent constipation; that is to say, to the congestion which was its natural consequence. Subject himself to hemorrhoids, though not habitually constipated, Dr. Riviere tried the podophyllin on himself, and found immediate relief. The hemorrhoids again made their appearance, but were removed by the same means. He has since frequently employed the same method of treatment, and always with equal success, giving one or two podophyllin pills of one-fifth of a grain each, so as merely to soften the fecal mass. Encouraged by this success, Dr. Riviere tried podophyllin for chronic hemorrhoids likely to necessitate operation. In these cases also he obtained immediate relief, and a cessation of all the usual distressing symptoms of this affection. The only drawback was the necessity for the daily administration of podophyllin. In some instances, however, the patients were able to leave off the use of the medicine for a long time. Some even were entirely cured.—*Druggists' Circular.*

**To Allay Itching.**

℞

Pulv. camphoræ,

Chloral hydrat, āā ʒ j.

Ung. aquæ rose, ℥ j. M.

The chloral and camphor are to be carefully rubbed together till a fluid results, and then the ointment is to be added slowly and well mixed. It does not answer when the skin is at all broken; the burning sensation caused on its first application lasts but a few moments, while the relief lasts for hours.

**Oil of Turpentine in Sciatica.**

A writer in the *Edinburgh Med. Jour.* recommends the following prescription in sciatica:

℞

Olei terebinthinæ, ʒ ij.

Olei ricini, ʒ iv.

Mucilaginis, ʒ iv.

Aquæ, ad ℥ ij. M.

The whole to be taken early in the morning. Several cases are given in detail, in which, after two or three trials of the remedy, the pain subsided.

**Belladonna in Constipation.**

Dr. H. A. DuBois, of San Rafael, Cal., writes:—"In a case of paralysis of the bowels, the result of apoplexy, when there had been no discharge for two weeks, and where Croton oil and other powerful cathartics had failed, and in which the ascending colon only could be emptied by the rectal tube, small doses—five drops three times a day—of the fluid extract of Belladonna, continued for two days, enabled the tube to enter the tranverse colon, and procured thorough action through the whole length of the canal. I was indebted to Dr. Taylor, U. S. Navy, for this suggestion."

**Lesions from Alcohol.**

An English contemporary justly remarks that immediate death from excessive drinking is a rare thing, and the number of such deaths is very little criterion as to the mortality caused by drink. Drunkenness kills not so much by sudden death, as by exciting comparatively slow processes of disease and degeneration, which appear in the mortality records under various names, but generally in no immediate association with the alcoholism which has largely caused them. Such processes are some forms of Bright's disease, of heart disease, of dropsy, of rheumatism, of vascular disease, of paralysis, etc. These degenerative processes are known by medical men to be common in men who drink, but are scarcely ever drunk.—*Med. and Surg. Reporter.*

**Mistura Guaiaci Viridis.**

℞

Potassii iodidi, ʒ iij.
Tr. guaiaci ammoniatæ, fl. ℥ ijss.
Aquæ, fl. ℥ iij.
Extracti fl. dulcamaræ, q. s. ad fl. ℥ viij

Dissolve the iodide of potassium in the water, add the tincture in portions, and shake well together until a clear green mixture has been produced, with separation of most of the gum-resin. Then strain, and add the fluid extract. Dose, in gout or rheumatism, one to two teaspoonfuls.

**Chronic Bronchitis.**

The administration of the following has been attended with a fair amount of relief in this class of cases:

℞

Oleum lini, ℥ ij.

Mucilagi acaciæ, ℥ ijss.

Syr. tolu, ℥ j.

Ol. terebinth, ʒ iij.

Aquæ cinnamomi, ad. ℥ viij.

M. S. ℥ss three times a day.

—*Druggists' Circular.*

**Croton Oil to the Skin.**

M. Ladriet de la Charriere recommends the following formula:

℞

Ol. theobromæ, 1 part.

Cera alba, 1 part.

Ol. Tiglii, 2 parts.

These ingredients are to be mixed together and moulded in the form of crayons, which may be preserved in tin foil. The oil thus prepared may be used in the treatment of tinea tonsurans or in other affections where the influence of croton oil is to be exercised over a limited area.

**Camphor-Vapor Baths.**

These baths are used with very good effect—so it is said, in the Queen's Hospital, Birmingham—for the relief of Bright's disease. They are also said to produce very free perspiration, and are given every evening in the following manner: The patient is seated upon a cane-bottomed chair, with a large blanket pinned around his neck. Half an ounce of camphor is placed upon a tin plate under the chair, and above the flame of a small spirit-lamp, by the heat of which the camphor is slowly vaporized. This plan of treatment is also employed in some cases of convalescence from acute or sub-acute rheumatism, when the action of the skin is defective, and when some pain and stiffness of the joints remain.—*New Remedies.*

**Fœcal Origin of Typhoid Fever.**

As the result of a most extensive study of typhoid fever, in which one hundred and six epidemics occurring in different parts of the world were analyzed, M. Jaccoud reaches the conclusion that fœcal matters engender the disease, but are not *typhogenic* unless they enclose the specific typhoid poison, which usually comes from the dejections of typhoid patients. There are circumstances, however, under which the fœcal matters are poisonous without having had any previous admixtures of typhoid matters; in such cases the poison is elaborated in the fœcal matters, which themselves are, as before, merely the agents of transmission.—*Journal de Medicine*, May, 1877.

**Hot-Water Injections in Uterine Hæmorrhages.**

In confirmation of the utility of this practice, as described by Dr. Runge, (*Medical Times and Gazette*, May 12, page 510), we may adduce the following short article, published by M. Ricord in the *Union Medicale*, June 5:—"Hæmorrhages in general, and metrorrhagias in particular, whatever may be their proximate cause, are, as is well known, very frequently difficult to arrest. Hæmostatics given internally, astringent injections of every description, plugging, &c., generally fail. But one means has almost had infallible success in my hands, viz:—the injection of hot water at 50° C, (122° Fahr.,) carried directly against the cervix uteri by aid of the tube of an irrigator from which the caoutchouc canula has been removed."

**The Poison of Small-Pox.**

In the session of the French Academy of April 30th, Messrs. Pasteur and Joubert made an important communication on the nature of the poison of small-pox (hæmorrhagic variety). They have succeeded in propagating the bacteria, which are contained in the blood during this disease, outside of the living organism, in "dead" liquids; and they found that their vitality remained unimpaired, even after repeated transplantation. Such an infected solution may be filtered by means of appropriate apparatus, and completely freed from bacteria, so as to become entirely inert. During the artificial propagation of these bacteria in clear solutions, no microscopic granulations appear to be formed, at least, even under the most powerful lens, no organized or amorphous substances can be traced outside of the bacteria. These facts make it highly probable that the poison of small-pox is a bacterion, and not a virus.—*Ber. d. Deutsch, Ch. G.*

**External Use of Chloral Hydrate.**

A writer in the *Canada Medical Record* recommends a solution of Chloral Hydrate in water, from three to five grains to the ounce, as a substitute for carbolic acid for external use. Its effect on ulcers is prompt and admirable, and a case of eczema was successfully treated by it. In cases of amputation, where the surfaces took on unhealthy action, the solution changed their character and removed the difficulty in twenty-four hours. It removes fetor, is clean and neat, does not stain, and stimulates granulations without destroying them.

Chloral hydrate has also been used in Germany as an antiseptic application in cancer of the uterus. A strong solution—one part to twelve of water—is applied by means of cotton. Dr. Goodell, of Philadelphia, also speaks highly of it as a purifying and deodorizing agent.

**Antiseptic Vinegar.**

The following formula for antiseptic vinegar is given by Pennes:

| | | |
|---|---|---|
| Salicylic acid, | 300 | grammes. |
| Acetate of alumina, | 300 | " |
| Tincture of eucalyptus globulus, | 1000 | " |
| " verbena, | 900 | " |
| " lavender, | 1000 | " |
| " benzoin | 100 | " |
| Acetic acid ("No. 8") | 1000 | " |

Mix thoroughly with the requisite precautions, shake frequently during two or three days, and filter.

This vinegar may be used either pure or diluted as a lotion.

Five litres injected into the carotid will preserve a cadaver several months. In the form of spray it is recommend as an admirable disinfectant for the sick-room.

**Cosmoline.**

In a recent number of *L' Union Medicale*, a report of a communication is given, made by Dr. G. Camust to the Society of Medicine of Paris, on the unctuous hydro-carbon obtained from petroleum, known as cosmoline. The petroleum oils of America, and the naphtha of the Caspian Sea and elsewhere, furnish, on distillation, a series of products, the lightest of which are gases, the formenic series, $C_2H_4$, and the heaviest the solid paraffines. Between them are the soft olefines. Cosmoline, with which M. Camusat experimented, he defines as unctuous, clear yellow, greasy, and adhesive, fusing at 40° Cent. It has no decided odor, a greasy taste, and a density of 0.92, between that of water and alcohol. It is not soluble in either of these, but is, in all proportions, in either. It is not affected by oxydizing agents, nitric or chromic acids, and is wholly neutral and without affinities. This inalterability makes it a valuable vehicle for local application; he had tried it with much satisfaction at the Hospital St. Louis. The chief use he had made of it was in ophthalmic surgery, as a pomade. It is perfectly well borne by the conjunctiva, and is superior, for application to such delicate parts, to anything Dr. Camusat has yet tried. For the general dressing of wounds, he had also found it a very superior article.—*Druggists' Circular.*

---

**Milk Diet in Amenorrhœa and Obesity.**

The *Medical Press and Circular* says:—Professor Tarnier has been induced to try milk diet as a remedy for the above complaints, from having observed the good effects produced in a patient suffering from Bright's disease, and being subsequently consulted by a young lady with albuminuria, great tendency to embonpoint, and with whom menstruation was arrested for some months. M. Tarnier's opinion was asked whether she was *enceinte*, but he found no symptoms of pregnancy, and advised the strict employment of a milk diet, as a remedy more for the albuminuria than for her sickness. He did not see his patient for some months, and when she consulted him again she presented the appearance of health, and had so diminished in size that he did not recognize her. She told him she had followed his prescription rigorously, experiencing rapid improvement; the urine lost its albumen, the excessive obesity disappeared, and as she grew thinner menstruation became re-established. Some time afterwards M. Tarnier was consulted by another lady, not suffering from albuminuria, but from obesity with arrested menses, and he advised a similar regimen. In her case, as in the preceding one, there was rapid improvement, the obesity disappeared, and menstruation returned. It is impossible to draw conclusions from two cases, but there can be no doubt of the efficacy of milk in the treatment of the albuminuria of pregnancy. M. Tarnier is very particular in the mode of administration of the milk, and requires from his patients the exact observance of the diet he lays down for them.

---

**Insomnia.**

M. Willemin, of Vichy, has recently published a paper on insomnia, which terminates with the following conclusions:

1. Morphine is the most powerfully hypnotic alkaloid of opium; narceine and codeine are less active, but do not leave behind the malaise that morphine produces. These substances are indicated in the insomnia of pain; they are counter-indicated when cerebral congestion exists.

2. Bromide of potassium, which is a much less powerful hypnotic, is indicated in insomnia with circulatory excitement, such as the nervous insomnia in which opiates often prove inefficacious. It is administered with success to children. It is counter-indicated in cases of very marked anæmia.

3. Sulphate of Quinine, like the bromide, seems to exert an action on the nervous elements, which is followed by diminution of the encephalic congestion; like these two drugs, chloroform administered by the mouth, proves useful, especially in nervous insomnia.

4. Hydrate of chloral is a pure hypnotic, and is more rapid in its action than the others. It is applicable in almost all forms of insomnia except in certain dyspnotic and cardiac affections, and when great debility exists.

5. The insomnia of old people and of weak, anæmic persons, may sometimes be combated with success by tonic medication, wine, ether, bitters and hydropathy.—*Lyon Medical*, June 17th.

---

**Subcutaneous Injection of Blood.**

Dr. Schmeltz, of Schlestadt, reports a case of consumption with great weakness, and intense anæmia, in which the subcutaneous injection of defibrinated blood was followed by the most satisfactory results. The patient was first seen by Dr. Schmeltz in March, 1874; he was then over sixty years of age, had been sick for a long time, and was confined constantly to his bed, in consequence of his extreme weakness. He was reduced almost to a skeleton, and almost all over his lungs there were dullness, bronchial breathing and moist rales. He had hectic, and suffered from various neuralgias, from frequent attacks of syncope, and from dyspnœa.

His stomach soon became weak, and neither food nor medicines could be retained. Dr. Schmeltz then determined to try subcutaneous injections of defibrinated blood, which had been first recommended by Dr. Karst, of Kreuznach. The blood used was taken by wet cups from the back of the patient's son and was carefully defibrinated. Eight injections of five grammes each were made into the arms and legs at one sitting, consequently forty grammes (ten and a quarter drachms) in all were injected. The swellings caused by the injections had disappeared at the end of the second day. The operation was followed by a very rapid improvement in the general condition of the patient. His appetite returned; the pulse became full and firm and 80 per minute; the neuralgias, anxiety, palpitations and extreme weakness were relieved, and he was able to sleep. Eight days after the operation he got up and convalescence was thenceforth uninterrupted. The patient is still living and in good health; during the last two years he has required no medical treatment.

Dr. Schmeltz thinks that the case proves that the hypodermic injections of blood may prove useful in many cases where transfusion is indicated, especially in cases of anæmia in which the stomach rejects all nourishment and medicine.—*Annals et Bulletin de Gand*, June, 1877.

---

**Changes of the Pupil in Chloroform Narcosis.**

In the surgical clinic in Gottingen during the past winter, the changes in the pupils during the administration of chloroform were carefully observed in one hundred and twenty-two cases. Previous to and during the stage of excitement, the pupils were in most of the cases of the usual width; in a few cases, just before the stage of complete insensibility, they were quite wide and sensitive to the light. During the stage of complete insensibility, they were closely contracted in one hundred and twenty of the cases, and were absolutely immovable in 119. An instantaneous dilitation of the pupils in this stage was found to be a threatening symptom of chloroform poisoning. This occurred in two of the cases, in one of which the trouble seemed to be located in the heart, and in the other in the lungs; in both, life was restored by pulling forward the jaw and resorting to artificial respiration.

The following practical lesson has been deduced from these observations: When, during the stage of tolerance, the pupils begin to dilate slowly, it is a sign that the patient is recovering from the narcosis and more chloroform must be given. When, on the other hand, the pupils become suddenly widely dilated, the administration of chloroform must be at once stopped, and further trouble guarded against—*Centralblatt fur Chirurgie*, June 23.

---

**The Dangers of Ether.**

It has always seemed to us the height of folly to declare there could be no danger in any anæsthetic. The lesson taught by a late death from nitrous oxide has, it is to be hoped, been well learned, and we shall in future hear less of the absolute safety of any agent capable of depriving a person of all sensation. Some cases in which ether has been followed by alarming symptoms have lately been recorded. They have been termed syncope, but the word is not appropriate, as the heart continued to beat after respiration ceased. This is what should have been anticipated. When death is produced by ether the animal's heart continues to beat long after the arrest of respiration. The pulse is quickened by ether and maintains its force through a long state of anæsthesia. In these facts lies the safety of ether. But it should never be forgotten that there is danger at a certain stage, and the danger is from the side of the respiration, which at length ceases. Stertorious breathing proceeds from the paresis of the muscles of the palate, and should lead to the ether being suspended. So respiration growing more and more shallow and less frequent is a warning, and should not be overlooked. It is very rare that the heart fails—perhaps never. Pallor is rare, too, and should excite attention if it occur. But, we repeat, the danger of ether is from the side of respiration, that of chloroform from the heart, and this fact goes far to explain their relative safety. In chloroform narcosis the danger is much more sudden. Ether gives warning.—*The Doctor* (London).

---

Some of the leading British medical journals, whose editors used to be unsparing in their denunciations of "Homœopathy," and of other journals for their publication of the advertisements of homœopathic schools, have quite modified their views, if we may judge by the appearance of their own advertising pages, *vide Lancet* and *Med. Times and Gaz.* Perhaps the action of the Vice-President of the British Homœopathic Medical Society may have had something to do with the change in their views of the question (see following item).—*New Remedies.*

HOMŒOPATHY IN ENGLAND.—"An exceedingly interesting correspondence on the subject of homœopathy is published in *The Lancet.* It shows a very different aspect of the matter in England from anything here. Dr. George Wyld is the Vice-President of the British Homœopathic Society. On the part of himself and associates he makes overtures asking whether there is no ground for reconciliation between the older school of medicine and his own. He says that British homœopathists have discarded their globules and infinitesimals, and give doses of tangible strength, sufficiently large to effect the object. They admit that Hahnemann's views were often extravagant and incorrect; they will accept the dictum of Hippocrates that some diseases are to be cured by similars, and some by contraries, and, "therefore," adds the Doctor, "it is unwise and incorrect to assume the title of homœopathist." *The Lancet* does not receive the overture in a liberal spirit, but assumes an air of arrogance, and lays down the rule, that British physicians can only receive homœopathists into the fold upon a full renunciation of their errors, and a complete acceptance of the ancient doctrine." This scant courtesy on the part of the regular practitioners will chill any tendency to similar concessions among homœopathists elsewhere."

---

**Pityriasis Capitis.**

In a paper read before the *Societe de Therapeutique* of Paris, reported in the *Bulletin General Therapeutique,* Dr. Martineau advocates the treatment of pityriasis capitis with solutions containing chloral. After remarking on the persistance of pityriasis and its obstinate resistance to the numerous drugs which have been tried against it, Dr. Martineau says, "If I am not deceiving myself, chloral offers us a means if not certain at least very efficacious for the treatment of the rebellious affection." In the hands of Dr. Martineau and also of Professor Tardieu, the following solution has given excellent results: water, 500 grammes; hydrate of chloral, 25 grammes. This solution should be made lukewarm, and applied in the morning with a sponge to the diseased parts. The part touched with it must not be wiped. If the pityriasis be recent, a single application will often suffice for its cure; if it be old, it disappears to re-appear later on. The solution of chloral always has the effect of causing a disappearance of the rash and the pruritus, so that it is sufficient to continue the lotion in a case of chronic pityriasis until the patient suffers no inconvenience from his disease. If the pityriasis be complicated with any other cutaneous affection, as erythema or prurigo, it is necessary, before employing the solution of chloral, to use the following liquid; water 500 grammes, hydrate of chloral, 25 grammes, Van Swieten's solution, 100 grammes. This solution should be used every morning with a small sponge. When the affection which complicates the pityriasis has disappeared, the chloral solution may be returned to. The application of solution of chloral causes immediate redness of the skin and provokes slight itching, but these inconveniences only last a few minutes.—*Druggists' Circular.*

---

**Salicine in Cancer of Stomach.**

Dr. E. A. Hull, of Berlin, N.Y., writes: "I wish to call attention to the great relief obtained in a case of carcinoma of the stomach from the use of salicine. The pain and vomiting was very severe, and the only way in which an anodyne (morphine), could be retained was to administer it in a state of effervescence, and then only for a short time; and under these circumstances the patient suffered greatly from the pain in the stomach and the annoyance of vomiting. At this stage I began giving small doses of salicine, perhaps half a grain every three hours, and to my surprise there was a marked relief from both the pain and the vomiting from the first powder, which was given in a little water and was perfectly retained in the stomach. Patient continued to take the salicine as long as life lasted, about five weeks, and enjoyed a degree of ease and comfort that was not to be anticipated. I am not aware that salicine has ever before been given for relief in this disease, neither do I pretend to say just what the *modus operandi* of the remedy was that gave such relief in this case, but think it must be owing to the anæsthetic and antiseptic properties of the salicylic acid contained in the salicine.

"As this is the only case in which I have had a chance to use the salicine in carcinoma of the stomach, I hope other physicians who may have an opportunity will give it a fair trial and report their results.

"The dose of salicine was increased some during the five weeks the patient took it, but at no other time did it exceed two grains."

---

**The Value of Frequently Repeated Small Doses of Medicine.**

Dr. H. S. Dessau recently read before the New York Medical Journal Association a paper of which the above title indicates its subject. Therapeutically considered it has more than ordinary value. The statements are not altogether new; it has been long known by some medical practitioners that there is great potency in small doses, and that large ones sometimes produce toxic rather than curative effects. Dr. Dessau has really done a service to the practice of medicine by giving prominence to some of the advantages of diminished doses, and shown that they should be frequently repeated, so that their effects may be sustained until a cure of the patient has been secured. He strongly disavows homœopathic tendencies. He is a believer in large, as well as minute, doses. He has seen the advantages of each. Nevertheless, it is certain, homœopathic practice has given a certain degree of shape to his opinions. Some of his instances, in which the efficacy of minimum quantities are recorded, have a familiar look of the system of similars.

The following quotations are an analytical abstract of the address. Facts are retained; the connecting matter is omitted. The complete paper was published in the *Medical Record*:—

RINGER'S HAND BOOK OF THERAPEUTICS.

Upon the appearance of that now indispensable little work, Ringer's "Handbook of Therapeutics," my attention was particularly attracted to the frequency with which he recommends small doses of medicines that we have been accustomed to use in much larger doses, for entirely different diseases. Some of these medicines were recommended so strongly that I was induced to give them a trial, more especially as my practice among children impels me, for many reasons, to administer as little unpleasant tasting medicines as possible. Their use with children having first been found satisfactory, my position in connection with the New York Dispensary afforded me the opportunity to further test their value in numerous cases of adults.

IPECAC. FOR VOMITING IN CHILDREN.

In the treatment of vomiting in children, whether due to stomach and intestinal disorder, or as a complication of pneumonia, following the recommendation of Ringer, I have found the administration of drop doses of the wine of ipecac, repeated every hour, act with the greatest success in checking the vomiting. It also appears to exert a curative effect upon the diarrhœa of children when attended with vomiting, especially that form where the stools resemble those of dysentery. But the vomiting is the symptom that is most markedly benefited.

FOWLER'S SOLUTION FOR THE VOMITING OF DRUNKARDS.

In the vomiting sometimes following a debauch, especially in women, of which I have seen several severe cases, drop doses of Fowler's solution of arsenic, hourly repeated, appeared to act like a charm. This remedy is also highly recommended by Ringer in the morning vomiting of drunkards, where this symptom is indicative of a chronic affection of the gastric mucous membrane.

ALUM FOR VOMITING IN PHTHISIS.

In the vomiting which often complicates phthisis pulmonalis and its allied affection, chronic bronchitis, independent of that brought on by the cough, it is of the utmost importance to be possessed of a reliable remedy to check it. It is almost astonishing to observe with what happy success small doses of alum, say from three to five grains, given in solution with some aromatic water, as cinnamon, for instance, act here. When the vomiting in these cases is frequent and severe, I have given the alum every second or third hour, but otherwise three times a day is sufficiently often. Rarely does the remedy need to be used beyond twenty-four hours. In a few cases that came under my observation, the vomiting, after having disturbed the patient for several days, has ceased after the second dose of the alum.

After some children have passed through an attack of pertussis, especially those of a lymphatic temperament, they are apt to be harassed with a troublesome cough for many months, which appears to be a slow winding up of the original disease.

TARTAR EMETIC IN BRONCHITIS OF CHILDREN.

There is a form of bronchitis seen amongst children, where a large number of coarse mucous rales produce loud wheezing with an asthmatic quality of cough. The wheezing is the symptom that the mother is most likely to complain of, and together with the cough is most intense at night, both almost entirely disappearing during the day. Such cases very readily yield in my practice under the use of tartar emetic, given in solution in the proportion of a grain to the pint of water. Of this solution a teaspoonful is given every one or two hours, with the best results, sometimes, according to Ringer, relieving the noisy wheezing after one or two doses.

Often in children we find a catarrh of the bronchial and intestinal mucous membranes, either coexisting or alternating with each other. When such a condition persists after the employment of the ordinary household remedies, tartar emetic in the same doses of the solution just before mentioned, hourly repeated, will check both catarrhs, without the use of further treatment.

MINUTE DOSES OF MERCURY IN SYPHILITIC HEADACHE.

In the treatment of syphilis we have come, as an almost universal rule, to rely upon the benefit of some form of mercury. That form most ordinarily in favor, in the earlier stages of the disease, is I doubt not the protiodide, and that in the more advanced stages the biniodide, in combination, perhaps, with the iodide of potassium. Special symptoms will arise, however, during the progress of such treatment, or present themselves primarily for treatment, that do not appear to be immediately influenced by either of the forms of mercury mentioned. Such, for instance, is the cephalalgia that I have seen rack and torment the patient in spite of large doses of chloral hydrate and bromide of potassium in combination, phosphorus, the hypodermic employment of the sulphate of morphia, or even free doses of iodide of potassium, the patient in the meanwhile undergoing a course of mercurial treatment after the plan above mentioned. The iodide of potassium may, however, afford relief from the pain after a certain duration of time, but perhaps not until after much exhaustion has been caused by the intense suffering.

It was after I had been almost baffled in dealing with such a case that I read in one of our journals, I think the *Medical News and Library*, an extract from a foreign journal upon the treatment of this complication in syphilis, by Dr. Peters, of Paris. He recommends the use of calomel in the one-sixtieth of a grain doses, every hour until the pain is relieved. A case presented itself in time and was a fair one to test the value of the treatment. No sleep had been obtained for three nights, and so great was the pain that there was complete anorexia. Before using a dozen of the powders, in which form the calomel was given, relief was obtained and sleep procured. In thirty hours the pain had entirely ceased, and no more powders were used. No signs of mercurial salivation were shown, and the appetite returned as soon as the pain was relieved.

Following the suggestions of Ringer, I have lately given preference to mercury with chalk, as the form of mercurial to administer in the summer diarrhœas of children. The usual dose is $\frac{1}{6}$ of a grain, given either with sugar or with three to five grain doses of subnitrate of bismuth, hourly repeated.

### CORROSIVE SUBLIMATE IN A CERTAIN VARIETY OF THE DIARRHŒA OF CHILDREN.

In a form of diarrhœa in children, likely to be mistaken for dysentery, but where the general symptoms are mild, and the special features are secondary to the diarrhœa, corrosive sublimate will be found to render most satisfactory service in effecting a cure. The principal indication for the use of corrosive sublimate, according to Ringer, is the mucous character of the stools, whether containing blood or not. There may also be more or less straining at stool. I have used the corrosive sublimate in such cases, in the proportion of a grain to sixteen ounces of water, which is half the strength recommended by Eustace Smith. Ringer recommends a grain to ten ounces of water. Of this solution, a teaspoonful is given every hour, or two hours, as the severity of the case demands.

### MINUTE DOSES OF ZINC IN GONORRHŒA.

Gonorrhœa may be said to be a specific catarrh of the urethra, where every practitioner has his own favorite remedy. This, of course, may depend upon the stage of the complaint, and other modifying circumstances. I here acknowledge my thanks to Prof. Ringer for his valuable suggestions on the treatment of this complaint, in his work so often quoted by me in this paper. When a case of gonorrhœa can be seen in the first twenty-four hours of the attack, as has been my good fortune in several instances, an injection of a solution of chloride of zinc, one grain to a pint of water, used every hour, will cut short the attack in twenty-four hours.

### COPAIBA IN URTICARIA.

About a year ago, under the head of "Notes of Hospital Practice," there appeared in the *New York Medical Journal* a paragraph on the value of small doses of copaiba in urticaria, as employed by me at the New York Foundling Asylum. Two cases, both of which were of a chronic nature, were especially cited to illustrate the beneficial results of this treatment. In these cases, to repeat, the affection had existed for two or three months, unsubdued by the usual treatment of salines and purgatives, and afterwards of arsenic and iron. It occurred to prescribe drop doses of copaiba three times daily. The theory of the treatment was founded upon a desire to test the value of the *similia similibus curantur* principle. It was a purely tentative treatment with me, though I have since been informed that the same treatment for urticaria had been noticed in some of our numerous medical journals previous to the date of my cases. I have not been able to find any journal containing any information on such subject, and therefore modestly lay claim to the originality of the treatment. I am willing to surrender my claim whenever a worthier claimant for priority presents himself. These cases yielded gratifying results, and since then I have treated numerous cases in children with a like success, as far as my knowledge of the results of those cases extends.

My main experience with the use of copaiba in urticaria has been amongst children, but I have had occasion to administer it in an acute attack in the adult. It was a severe one, following the eating of lobster-salad. It had persisted in its severity for three days and nights, before I began the use of the copaiba. Purgatives, saline diuretics and mustard foot-baths had been freely given in the meantime, without any perceptible improvement of the eruption. Drop doses of the copaiba were then given every hour, and in eight hours there was a marked diminution of the eruption, and the next morning it had entirely disappeared. It may have been that this result would have occurred spontaneously, but following so closely upon the use of copaiba as it did, it appeared to me at least a remarkable coincidence, that justifies further trials of a similar treatment in future cases.

ERGOT IN RETARDED MENSTRUATION.

Two cases of retarded menstruation, occurring in healthy females, non-pregnant, and ordinarily regular, have been treated by me with drop doses of fluid extract of ergot, hourly repeated. The menses appeared within twelve hours after treatment was commenced. In one of the cases the same treatment was employed a second time, one year after the first, with a like success.

WITCH HAZEL IN EPISTAXIS.

In three cases epistaxis of a severe degree—one apparently due to vicarious menstruation in a young negress, the second probably due to a lesion of the cardiac valves, in a small girl, and the third likely complicated with the hemorrhagic diathesis, in a male adult—marked benefit was derived from the use of fluid extract of hamamelis given in five-drop doses three times daily. Ringer recommends drop doses of hamamelis to be given every hour until the bleeding is checked, and then continuing it for a few days in five-drop doses, three times daily.

ACONITE IN REDUCING TEMPERATURE.

Aconite receives the highest recommendation especially for the purpose of reducing temperature and checking inflammatory processes, from both Ringer and Bartholow. The latter speaks of this medicine as a powerful agent which will produce manifest results in small doses.

BELLADONNA FOR SORE THROAT AND ACUTE ERYSIPELAS.

Bartholow pronounces strongly in favor of belladonna as a cure for idiopathic erysipelas, especially when affecting the face. He also remarks its prompt action in acute nasal catarrh with profuse watery secretion, and in ordinary sore throat. He advises giving five drops of the tincture as a first dose, and repeating with one or two drops every hour. Hughs regards belladonna as displaying wonderful powers in catarrhal throat affections.

NUX VOMICA FOR SICK HEADACHE.

Tincture of nux vomica also appears, according to Ringer, to be possessed of real curative powers, when given in drop doses, repeated every five or ten minutes for eight or ten doses, and then continued at intervals, for "sick headache," accompanied with acute gastric catarrh, whether due to error of diet, constipation, or no apparent cause. He regards it, administered in small and frequently repeated doses, as useful in many disturbances of the gastric functions.

Many other medicinal agents are mentioned by Ringer, Bartholow, and other writers, as being valuable and reliable in small and frequently repeated doses, in the treatment of various disorders. My friend, Dr. Geo. H. Fox, has informed me that he has witnessed decided beneficial effects from the use of small doses of pulsatilla in painful menstruation. Many of us have no doubt long been familiar with the use of small doses of castor oil in certain forms of diarrhœa in children. An every-day practice, that has become so common as perhaps not to attract our attention to the fact, is the large use of mineral waters for the cure of various dyspeptic and renal complaints. Here the actual dose of the supposed remedial salts has been shown to be quite small, the only one taken in any appreciable quantity being chloride of sodium, of which we use more in our daily food than is contained in a pint of most mineral waters.

Dr. Edward Vanderpoel, in an article on strychnia in tetanus and hydrophobia, published in the *Medical and Surgical Reporter*, of May 7, 1870, refers to eight cases of tetanus, seven traumatic and one idiopathic, coming under his care, which he cured with rather large doses of strychnia, giving $\frac{1}{6}$ to $\frac{1}{2}$ of a grain every two hours until relaxation of the muscles took place, when the interval was extended to every six hours. He states that he published full reports of those cases in the *New York Journal of Medicine*, Nov. and Jan. Nos. for 1846 and 1847.

If I am asked to explain on what principle these small doses act in certain diseases, I reply, on the principle, so far as known, of actual experience (!) This is all we know about it. The homœopaths claim that it is explained by the law of similars, which is no explanation at all; and they pretend to make universal application of this so-called law in the treatment of disease. Trousseau, and Bartholow following him, attribute to it a substitutive action, or, as the latter writer expresses it, the therapeutical action is the physiological antagonist of the disease action. It will, I think, be found that the necessity for the frequent repetition of the small dose will be in direct ratio to the acute or chronic character of the complaint. Hourly doses will be best indicated in acute cases both to impress the disease quickly and maintain the effect of the remedy; while in chronic cases a more chronic treatment is advisable.—*Druggists' Circular.*

